# Valine induces inflammation and enhanced adipogenesis in lean mice by multi-omics analysis

**DOI:** 10.3389/fnut.2024.1379390

**Published:** 2024-05-13

**Authors:** Hui-Yi Zheng, Li Wang, Rong Zhang, Ran Ding, Cai-Xia Yang, Zhi-Qiang Du

**Affiliations:** ^1^College of Animal Science and Technology, Yangtze University, Jingzhou, Hubei, China; ^2^Center of Animal Breeding Technology Innovation of Hubei Province, Wuhan, China

**Keywords:** adipose tissue, metabolite, metagenome, small intestine, valine

## Abstract

**Introduction:**

The branched-chain amino acids (BCAAs) are essential to mammalian growth and development but aberrantly elevated in obesity and diabetes. Each BCAA has an independent and specific physio-biochemical effect on the host. However, the exact molecular mechanism of the detrimental effect of valine on metabolic health remains largely unknown.

**Methods and results:**

This study showed that for lean mice treated with valine, the hepatic lipid metabolism and adipogenesis were enhanced, and the villus height and crypt depth of the ileum were significantly increased. Transcriptome profiling on white and brown adipose tissues revealed that valine disturbed multiple signaling pathways (e.g., inflammation and fatty acid metabolism). Integrative cecal metagenome and metabolome analyses found that abundances of *Bacteroidetes* decreased, but *Proteobacteria* and *Helicobacter* increased, respectively; and 87 differential metabolites were enriched in several molecular pathways (e.g., inflammation and lipid and bile acid metabolism). Furthermore, abundances of two metabolites (stercobilin and 3-IAA), proteins (AMPK/pAMPK and SCD1), and inflammation and adipogenesis-related genes were validated.

**Discussion:**

Valine treatment affects the intestinal microbiota and metabolite compositions, induces gut inflammation, and aggravates hepatic lipid deposition and adipogenesis. Our findings provide novel insights into and resources for further exploring the molecular mechanism and biological function of valine on lipid metabolism.

## Introduction

The essential branched-chain amino acids (BCAAs: leucine, isoleucine, and valine) supplied in dietary protein are of functional importance to the metabolic health of mammals (1, 2). In metabolic diseases, such as obesity and diabetes, the level of BCAAs is increased and becomes a circulating hallmark of insulin resistance (2–5). As BCAAs accelerate lipid deposition in metabolic tissues (6), dietary protein restriction was proposed recently to reestablish metabolic health, through multiple nutrient-sensing signaling pathways, especially the mammalian target of the rapamycin complex 1 (mTORC1) pathway (7–9). However, the exact role of BCAAs in health and lifespan remains conflicting (10, 11).

BCAAs have been examined originally on their systematic effects and biological and physiological functions, focusing on metabolic organs. In brown adipose tissue, BCAAs were found to be transported into mitochondria by SLC25A44 and utilized for heat production (12). When cross-talk between the BCAA metabolism and the mitochondrial pyruvate carrier is inhibited, the plasma BCAA level is reduced by activating the mTOR axis (13). During insulin resistance (IR), the BCAA flux is affected by the adipocyte-specific neutral amino acid transporter SLC7A10 to enhance lipogenesis and elevate the circulating level of valine-derived metabolite 3-hydroxyisobutyrate (3-HIB) (14). Recent studies shift to the separate or specific role of each BCAA in adipogenesis and systemic metabolism. Leucine can relieve IR and the browning of white adipose tissue, but isoleucine showed contradictory results (15, 16). Isoleucine is a key regulator in the adverse metabolic response to BCAA (17), and its restriction increases the health span and longevity in mice (18). As for valine, it is detrimental to glucose uptake and IR, with negative impacts on metabolic homeostasis (19, 20). However, functional results on valine in humans and mice were largely obtained based on high-fat diet (HFD) feeding or obesity models. Without HFD intervention or in lean animals, valine is also demonstrated to be vital to growth, immunity, and reproduction (21, 22). We previously discovered that valine affected the tissue structure and induced apoptosis in mouse testis (23). However, the molecular mechanism underlying the detrimental effect of valine on metabolic health remains less explored.

The integrative omics method can be effectively and efficiently employed to study the molecular changes underlying complex traits of agricultural importance (24–26) and also the biochemical and physiological function of bioactive substances (27). In particular, the combined metagenome and metabolome analysis can help understand the intricate interaction between microbe and metabolite, and their involvement in BCAAs biosynthesis and metabolism. In humans with IR, the main bacterial species (*Prevotella copri* and *Bacteroides vulgatus*) can impact BCAA biosynthesis and augment circulating BCAA levels (28, 29). Metabolome analysis found that BCAA supplementation significantly increased the oxidation of valine, but not that of leucine (30). The intestinal mucosal integrity is compromised by BCAAs, and the microbial composition is also disturbed by valine (31). However, the relationship between the specific detrimental effect of valine on metabolic health and the composition of gut microbiome and metabolites in lean mice remains unknown (21).

In order to explore the molecular mechanism underlying the detrimental effect of valine on metabolic health, in the present study, we employed first transcriptome sequencing on both white and brown adipose tissues and then combined cecal metagenome and metabolome analyses on lean mice treated with L-valine. We identified that valine induced inflammation and compromised structure integrity of the small intestine and enhanced adipogenesis and hepatic lipid deposition. Moreover, for the composition of microbial species, *Bacteroidetes* were decreased, but those of *Proteobacteria* and *Helicobacter* were increased.

## Materials and methods

### Ethics statement

Animal care and experimental procedures were conducted in accordance with the Animal Research Committee guidelines of Yangtze University, Hubei Province, China (YZU-2018-0031).

### Animals and dosage information

KunMing (KM) mice (male, 3-week-old) were purchased from the China Three Gorges University Experimental Animal Center [Yichang, China, SCXK (E)2022–0012]. Mice were kept in the independent ventilation cages (IVC) system (Suhang Tech, Suzhou, China) (24 ± 2°C, humidity 50–60%) on a 12 h:12 h light–dark cycle. After 1-week of adaptation, mice (body weight range: 23.97 ± 1.33 g) were randomly divided into three groups (eight mice per group and four mice per cage): chow diet (control or 0 V), chow diet +0.30% L-valine (w/v) (0.3 V), and chow diet +0.45% L-valine (0.45 V). The mice fodder formula is shown in Supplementary Table S1. L-valine (purity >99%, V0010, Solarbio and Aladine, China) was supplemented to mice via drinking water (w/v) and made freshly every 3 days. We defined the doses of L-valine in preliminary experiments and used the same protocol to examine the effect of valine on mice testis development as published recently (23). All mice showed normal growth rates, and no death of animals occurred during all experiments. Mice in different groups were caged separately and treated for 30 days, with free access to a chow diet and drinking water. For each group, the amount of water and feed intake were measured each day and divided by four to get a value for each mouse. The body weights of each mouse were measured every 3 days. Stool samples of mice were collected and stored at −80°C for subsequent experiments.

### Tissue samples

After treatment for 30 days and fasting for 12 h, mice were euthanized by cervical dislocation, and the blood samples were collected and centrifuged in heparin tubes. The tissue samples of the liver, adipose tissues [epididymal WAT, interscapular BAT, and inguinal beige adipose tissue (BeAT)], the gut, and different segments of the small intestine (the duodenum, the jejunum, the ileum, and the cecum) were excised, and relevant phenotypic measures were collected. All samples collected were immediately stored at −80°C.

### Histology

For histological analysis, the liver, the duodenum, the jejunum, and the ileum tissues collected for mice in each group were fixed in the 4% paraformaldehyde at room temperature overnight and then embedded in paraffin and sliced into 5 μm sections. The sections were dehydrated with 75, 85, 95, and 100% ethanol, sealed with Neutral Balsam (G8590, Solarbio) after transparent xylene, followed by the hematoxylin and eosin staining (HE staining kit, G1120, Solarbio). Slides were finally visualized, and images, villus height, and crypt depth of the small intestine were observed under an inverted microscope (OLYMPUS, IX73, Japan).

### Biochemical assays

The levels of triglycerides (TG) and total cholesterol (TC) in the serum and the liver were determined using commercial assay kits (Nanjing Jiancheng Bioengineering Institute, Nanjing, China) according to the instructions of the manufacturer.

### Adipose tissue transcriptome sequencing

Transcriptome sequencing (RNAseq) included a total of 12 samples: for epididymal white adipose tissue (WAT), three controls (CW1–CW3) and three from the 0.45% valine group (VW1–VW3) were included; for interscapular brown adipose tissue (BAT), three controls (CB1–CB3) and three from the 0.45% valine group (VB1–VB3) were included. Total RNAs for white and brown adipose tissues were extracted using 1 mL TRIzol Reagent (Invitrogen Life Technologies, Carlsbad, CA, United States) according to the manufacturer’s instructions. After quality control (Supplementary Table S2), a total of 3 μg RNA per sample was used as the input materials for transcriptome sequencing (RNAseq). Sequencing libraries were constructed by the NEBNext^®^ Ultra™ Directional RNA Library Prep Kit for Illumina^®^ (NEB, United States) following the recommendations of the manufacturer. Library quality was assessed on the Agilent Bioanalyzer 2,100 system and was sequenced on the Illumina NovaSeq 6,000 platform to generate 150-bp paired-end reads.

Clean reads were obtained by removing raw reads containing adapter and N ratio greater than 0.002, and the reads with low-quality bases exceeding 50% of the length ratio of the reads in single-ended reads, by fastp-0.23.2. After filtering, the sequencing error rate (Q20 and Q30) and GC content distribution were calculated. Reference genome and transcript annotation files (GRCm39 Ensembl release 107) were downloaded directly from Ensembl.^1^ Bowtie2 and TopHat v2.0.9 were used to align the paired-end clean reads to the reference genome (32, 33). The mapped reads were assembled by Cufflinks (v2.1.1) in a reference-based approach (34), and genes with zero expression levels in each sample in the matrix were deleted. Deseq2 (1.36.0) was used to determine transcripts or genes with a *P*-adjust<0.05 and |log2foldchange|>1 to be significantly differentially expressed, after gene expression normalization. Gene ontology (GO) and Kyoto Encyclopedia of Genes and Genomes (KEGG) were used for gene enrichment analysis and functional annotation by clusterProfiler package in R (the significance threshold set at *P*-adjust<0.05).

### RNA extraction and gene expression analysis

The total RNA of the liver, adipose tissues (the WAT, the BAT, and the BeAT), and the small intestine (the duodenum, the jejunum, and the ileum) tissues were extracted using the TRIzol reagent (Takara, Japan) according to the instructions of the manufacturer. Reverse transcription was performed, and qRT-PCR was carried out on a 7,500 Real-Time PCR System (Applied Biosystems, Carlsbad, CA, United States), using the Roche FastStart Universal SYBR Green Master Mix (Roche Molecular Systems, Basel, Switzerland). β-Actin was chosen as the internal control and analyzed using the 2^−ΔΔCT^ method (primer sequences are provided in Supplementary Table S3).

### Cecal metagenome sequencing

A total of six samples (control: C1-3, 0.45% valine: V1-3) were selected for shotgun metagenomics sequencing. According to the routine process of Beijing Novogene Technology Co., Ltd. (Beijing, China), the raw data obtained from the Illumina HiSeq sequencing platform using Readfq (V8, https://github.com/cjfields/readfq) were processed to obtain the clean data for subsequent analysis. The specific steps were as follows: (a) Remove reads with low-quality bases (default quality threshold is <38) that exceed a certain proportion (default length is 40 bp); (b) Remove reads with N bases reaching a certain proportion (default length is 10 bp); and (c) Remove reads whose overlaps with adapters exceed a certain threshold (default length is 15 bp). Considering the possibility of host contamination in samples, clean data needed to be BLASTed to the host database to filter out reads that may come from host origin. MEGAHIT software (v1.0.4-beta) was used for assembly analysis of clean data, with assembly parameter settings:--presets meta-large (−-end-to-end,--sensitive,-I 200,-X 400), and Scaftigs without N were obtained by breaking the resulted Scaffolds from the N junction. Clean data of each sample were aligned to the initial gene catalog by using Bowtie2 (Bowtie2.2.4) to calculate the number of reads of the genes on each sample alignment, with parameter settings: --end-to-end,--sensitive,-I 200, and -x 400. Genes with reads≤2 in each sample are filtered out to finally determine the gene catalog (Unigenes) for subsequent analysis.

DIAMOND software (V0.9.9.110, https://github.com/bbuchfink/diamond/) (35) was used for the alignment of Unigenes sequences with those of bacteria, fungi, archaea, and viruses extracted from the NR database of NCBI (Version 2018-01-02, https://www.ncbi.nlm.nih.gov/), with parameter settings:-blastp,-e 1e-5, and Evalue≤min. Evalue *10 of each sequence was selected. Meanwhile, DIAMOND software was used to align Unigenes with those in the functional database, with parameter settings: blastp,-e 1e-5. Functional databases include the KEGG database (Version 2018-01-01) (36), the eggNOG database (Version 4.5) (37), and the Carbohydrate-Active EnZymes (CAZy, Version 201,801) database (38). From the alignment results of each sequence, the best Blast hit results were selected. MetaStat and linear discriminant effect size analysis (LEfSe) were used to count the differential bacteria and metabolic pathways between the two groups.

### Untargeted metabolomics

A total of 12 samples (control: C1-6, 0.45% valine: V1-6) were selected for untargeted metabolomics. According to the process of Beijing Novogene Technology Co., Ltd. (Beijing, China), UHPLC–MS/MS analyses were performed using a Vanquish UHPLC system (Thermo Fisher, Germany), coupled with an Orbitrap Q ExactiveTM HF mass spectrometer (Thermo Fisher, Germany). The raw data files generated by UHPLC–MS/MS were processed using Compound Discoverer 3.1 (CD3.1, Thermo Fisher) to perform peak alignment, peak picking, and quantitation for each metabolite.

The metabolites were annotated using the KEGG database,^2^ the HMDB database,^3^ and the LIPID MAPS database.^4^ Principal components analysis (PCA) and partial least squares discriminant analysis (PLS-DA) were performed to verify the quality of the obtained data by metaX (39). The metabolites with VIP > 1 and a *p*-value of <0.05 and a fold change of ≥1.2 or an FC of ≤0.833 were considered to be differential metabolites. Volcano plots were produced by ggplot2, and the clustering heat maps were plotted by the Pheatmap package in R language. The functions of these metabolites and metabolic pathways were studied using the KEGG database after the enrichment of differential metabolites.

### Combined metagenomics and metabolomics analysis

The top 10 differential bacteria in the metagenome and the top 20 differential metabolites in the metabolomic were selected for correlation analysis. Pearson’s correlation coefficient R was calculated (scipy.stats.pearsonr in python SciPy) between the relative abundance of each different genus and the quantitative values of different metabolites at the genus level. The heatmap of correlation analysis was drawn by using the corrplot package in R.

### Elisa

Levels of adenosine 5′-monophosphate (AMP)-activated protein kinase (AMPK), pAMPK, and stearoyl-coenzyme A desaturase 1 (SCD1) (Enzyme-linked Biotechnology Co., Ltd., Shanghai, China) in the hepatic and adipose tissues, and two metabolites, stercobilin (Cloud-Clone Corp., Houston, United States) and indole-3-acetic acid (3-IAA) (Enzyme-linked Biotechnology Co., Ltd., Shanghai, China), in the small intestine (the duodenum, the jejunum, and the ileum) and stool, were assessed using commercial ELISA kits, respectively.

### Statistical analysis

Data analyzed by Student’s *t*-test, a one-way analysis of variance (ANOVA), and the least significant difference (LSD) for gene expression, protein, and metabolite abundances were expressed as mean ± SEM. Statistical analysis was performed using Prism 5.0 (GraphPad Software, Inc.) and SPSS Statistics 25 (IBM) software.

## Results

### Valine reorganizes hepatic and intestinal structure

**Figure 1:**
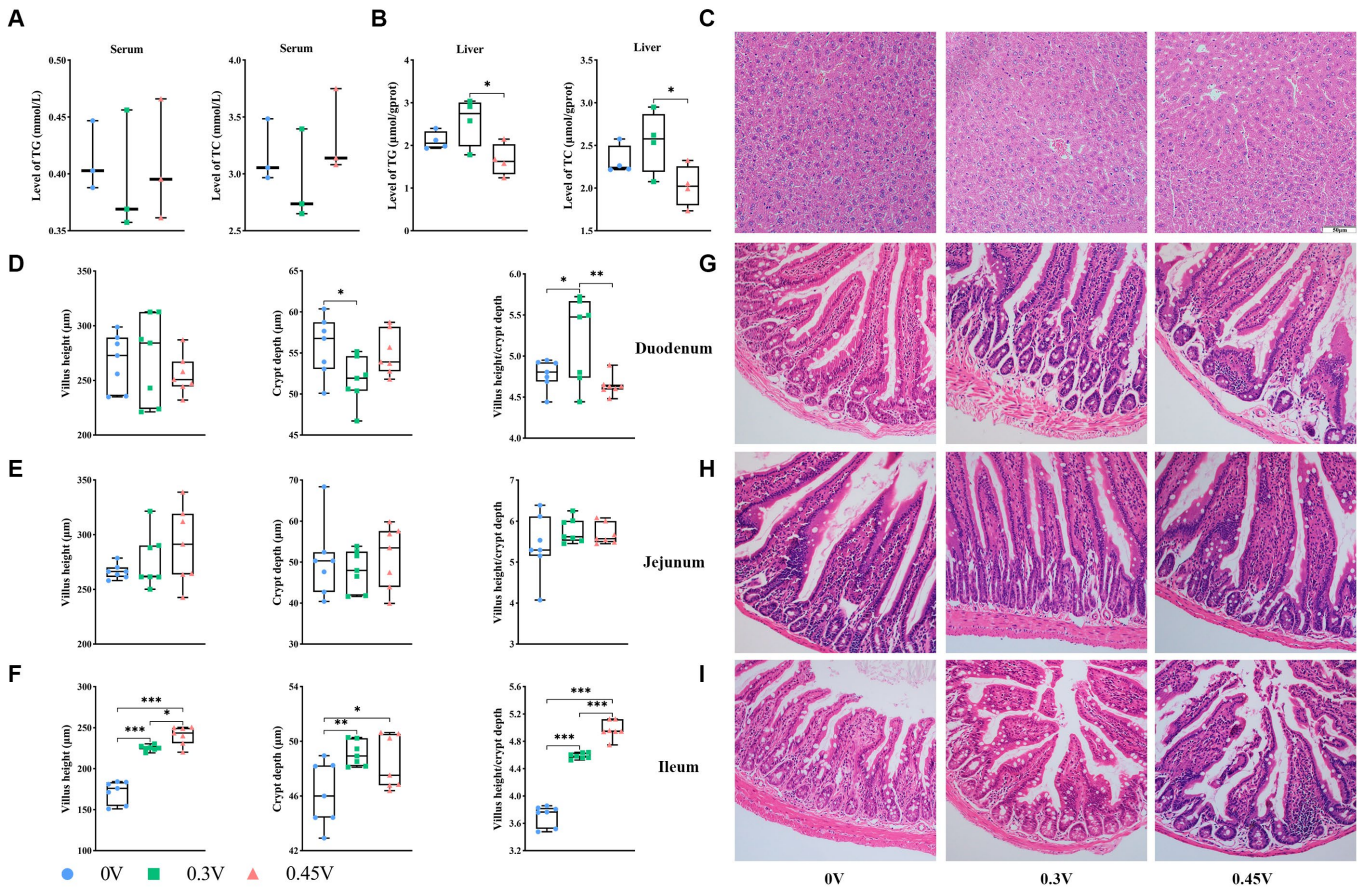
Disorganization of hepatic and intestinal tissue structure by valine treatment. TG and TC levels in **(A)** serum; and **(B)** liver; **(C)** hepatic histology staining; the villus height, crypt depth, villus/crypt ratio, and HE staining for **(D)** and **(G)** duodenum; **(E)** and **(H)** jejunum; **(F)** and **(I)** ileum. ^*^p < 0.05, ^**^p < 0.01, ***p < 0.001.

The detrimental effect of valine on metabolic homeostasis was mainly demonstrated in HFD-treated or obese animal or human subjects (19–23). Thus, we began with the experiment on the effect of valine on the growth and metabolism of lean mice. No significant differences were observed for the body weight and daily growth rate (Supplementary Figures S1A,B) and also for the feed and water intake (Supplementary Figures S1C,D) between the 0 V and valine treatment (0.3 V and 0.45 V) groups. After tolerance tests for glucose (GTT), pyruvate (PTT), and insulin (ITT) (Supplementary Figures S1E–G), it showed that mice treated with valine were slightly less sensitive to insulin. Furthermore, we assayed the levels of TG and TC. Significant differences did not exist in serum (Figure 1A). However, in the liver, both TG and TC levels were different between the 0.3 V and 0.45 V groups (*p* < 0.05) (Figure 1B).

Histology staining on the liver and small intestine tissues showed that compared to the 0 V group, 0.3 V and 0.45 V groups disturbed the hepatic structure, with more disoriented and scarce hepatic cords, narrower sinusoid, and more vacuoles (Figure 1C). As for the small intestine, valine treatment significantly reduced the crypt depth for the duodenum (*p* < 0.05) (Figures 1D,G), but without effect on the jejunum (Figures 1E,H). Notably, for the ileum, the villus height (*p* < 0.05 or *p* < 0.001), crypt depth (*p* < 0.05 or *p* < 0.01), and their ratio (*p* < 0.001) were increased between the 0 V and valine treatment (0.3 V and 0.45 V) groups (Figures 1F,I).

### Valine enhances hepatic and adipose lipid deposition

**Figure 2:**
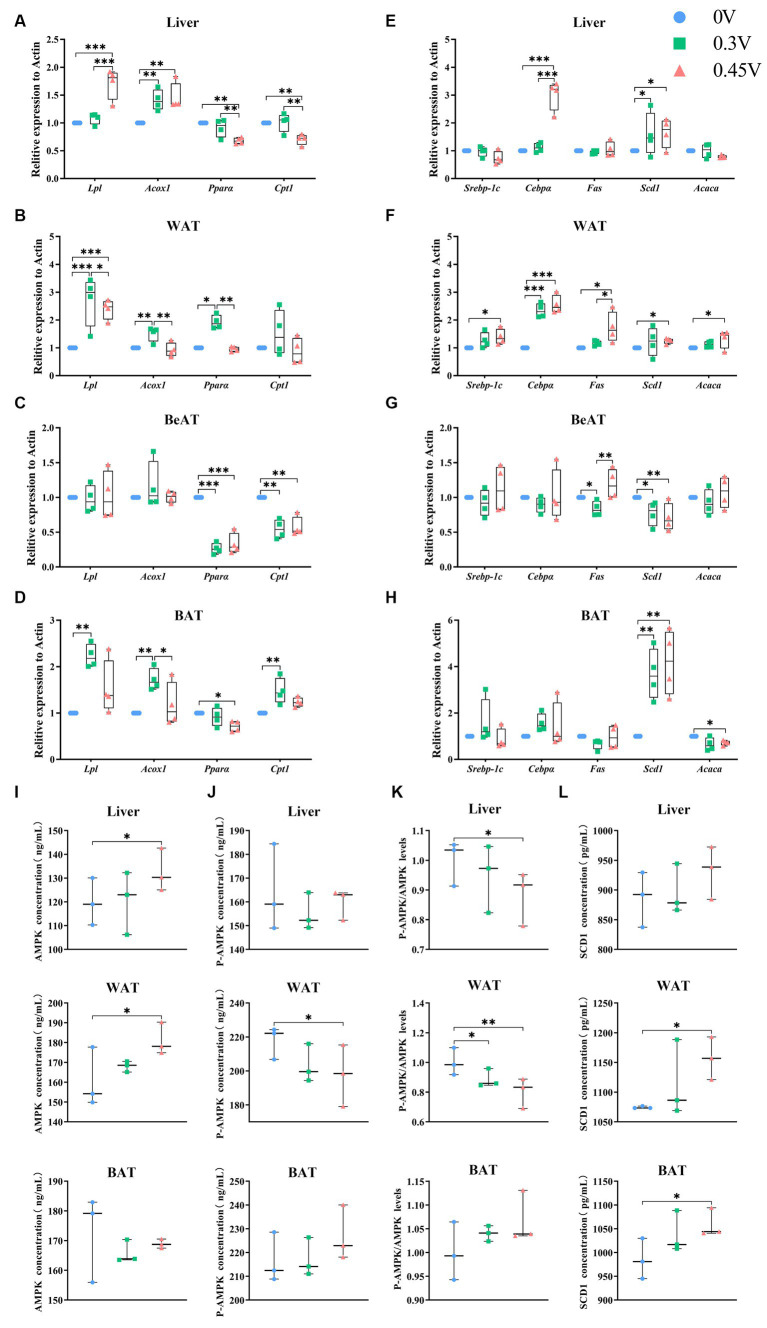
Enhanced hepatic lipid deposition and adipogenesis by valine. Expression profiling on genes related to β-oxidation and adipogenesis. **(A,E)** Liver; **(B,F)** WAT; **(C,G)** BeAT; **(D,H)** BAT; Protein abundances of **(I)** AMPK; **(J)** p-AMPK; **(K)** p-AMPK/AMPK ratio; **(L)** SCD1 in the vertical panel, for the liver (up), WAT (middle), and BAT (down), respectively. ^*^p < 0.05, ^**^p < 0.01, ^***^p < 0.001.

Next, we profiled the expression levels of genes related to β-oxidation [lipoprotein lipase (*Lpl*), acyl-coenzyme A oxidase 1 (*Acox1*), peroxisome proliferator-activated receptor α (*Pparα*), and carnitine palmitoyltransferase 1 (*Cpt1*)] and adipogenesis [CCAAT/enhancer binding protein α (*Cebpα*), *Scd1*, sterol regulatory element binding transcription factor 1 (*Srebp-1c*), fatty acid synthase (*Fas*), and acetyl-CoA carboxylase 1 (*Acaca*)] in hepatic and adipose tissues. For β-oxidation genes (Figures 2A–D), LPl and *Acox1* were enhanced in the liver (*p* < 0.01 or *p* < 0.001), WAT (*p* < 0.05 or *p* < 0.01 or *p* < 0.001), and BAT (*p* < 0.05 or *p* < 0.01) for 0.3 V and 0.45 V groups, except for the BeAT. *Pparα* was inhibited in the liver (*p* < 0.01), BeAT (*p* < 0.001), and BAT (*p* < 0.05) for the 0.45 V group, but elevated in WAT (*p* < 0.01) for the 0.3 V group. Similarly, *Cpt1* was inhibited in the liver (*p* < 0.01) and BeAT (*p* < 0.01) for the 0.45 V group but enhanced in BAT (*p* < 0.01) for the 0.3 V group.

As for adipogenesis genes, they were all significantly enhanced in WAT (*Cebpα*, *p* < 0.01; *Scd1*, *Srebp-1c*, *Fas* and *Acaca*, *p* < 0.05) for the 0.45 V group. *Scd1* was disturbed in all tissues for the 0.45 V group, enhanced in the liver (*p* < 0.05), WAT (*p* < 0.05), and BAT (*p* < 0.01), but inhibited in BeAT (*p* < 0.01), respectively (Figures 2E–H). For the other genes, *Cebpα* was elevated in the liver (*p* < 0.001) and WAT (*p* < 0.001) of the 0.45 V group (Figures 2E,F); *Fas* was elevated in WAT (*p* < 0.05) of the 0.45 V group (Figure 2F); *Acaca* was elevated in WAT (*p* < 0.05) but inhibited in BAT (*p* < 0.05) of the 0.45 V group, respectively (Figures 2F,H).

The protein abundances of AMPK, pAMPK, and SCD1 were further assayed in the liver, WAT, and BAT. As AMPK was elevated in the liver and WAT (*p* < 0.05) (Figure 2I) and pAMPK was reduced in WAT (*p* < 0.05) (Figure 2J), leading to the decrease in the pAMPK/AMPK ratios (*p* < 0.05, liver; *p* < 0.01, WAT) (Figure 2K). In WAT and BAT, SCD1 abundances were all increased (*p* < 0.05) (Figure 2L), consistent with its transcription levels.

### WAT and BAT transcriptome sequencing

As lipid deposition in the liver and adipose tissues was affected by valine treatment (0.45 V), we next performed WAT and BAT transcriptome sequencing (RNAseq), to check whether valine reprogrammed the global transcriptome. Raw reads were filtered to obtain clean reads of good quality for downstream analysis (Supplementary Table S4). The distribution of gene expression levels showed the transcriptional dynamics for each sample (Supplementary Figures S2A,B), and after PCA, samples could be clustered into two separate groups for WAT and BAT, respectively (Supplementary Figures S2C,D).

**Figure 3:**
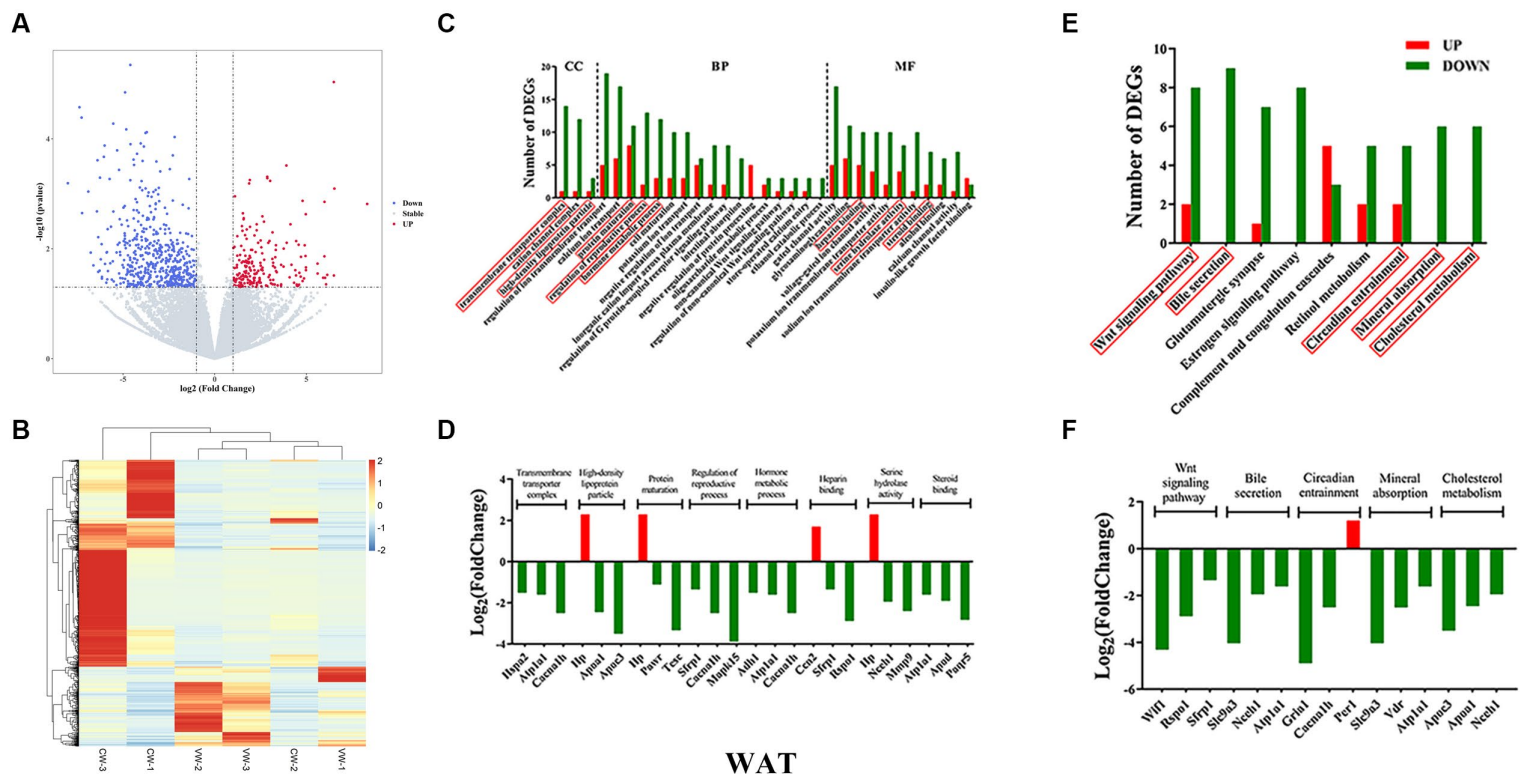
White adipose tissue transcriptome sequencing. **(A)** volcano plots; **(B)** heatmap of DEGs; **(C)** gene ontology (GO); **(D)** top three genes in each GO term; **(E)** KEGG pathways; **(F)** top three genes in each signaling pathway.

**Figure 4:**
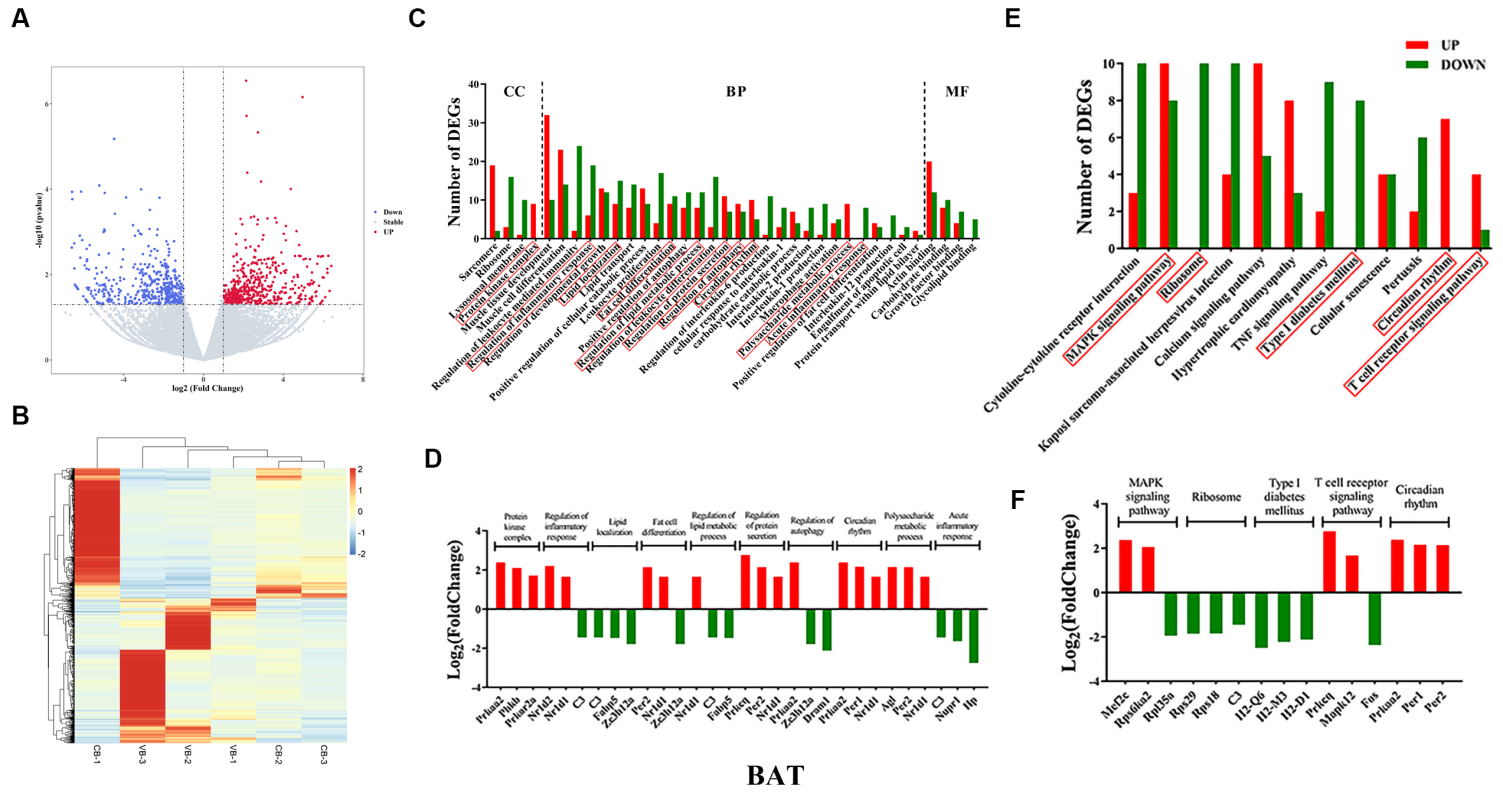
Brown adipose tissue transcriptome sequencing. **(A)** volcano plots; **(B)** heatmap of DEGs; **(C)** gene ontology (GO); **(D)** top three genes in each GO term; **(E)** KEGG pathways; **(F)** top three genes in each signaling pathway.

After multiple significance testing, 740 and 980 significantly differentially expressed genes (DEGs) were found for WAT (up, 534; down, 206) and BAT (up, 517; down, 465), respectively (Figures 3A,B, 4A,B). For WAT, gene enrichment analysis based on DEGs found the following GO terms (CC: transmembrane transporter complex, high-density lipoprotein particle; BP: protein maturation, regulation of reproductive process, hormone metabolic process; MF: heparin binding, serine hydrolase activity, steroid binding) (Figure 3C). The top-ranked DEGs (normalized counts ≥10, framed in red for CC, BP, and MF) were mostly downregulated, except for *Hp* and *Ccn2* (Figure 3D). For BAT, the GO terms were enriched as follows (CC: protein kinase complex; BP: regulation of inflammatory response, lipid localization, fat cell differentiation, regulation of lipid metabolic process, regulation of protein secretion, regulation of autophagy, and circadian rhythm; MF: carbohydrate binding) (Figure 4C). The top-ranked DEGs in protein kinase complex, regulation of protein secretion, circadian rhythm, and carbohydrate binding were upregulated, whereas those in the regulation of inflammatory response and lipid localization were downregulated (Figure 4D).

KEGG pathway analysis was then performed. For WAT, the WNT signaling pathway, bile secretion, circadian entrainment, mineral absorption, and cholesterol metabolism were found (Figure 3E). Nearly all DEGs were downregulated, except for the *Per1* gene (Figure 3F). For BAT, the MAPK signaling pathway, ribosome, type I diabetes mellitus, circadian rhythm, and T-cell receptor signaling pathway were found (Figure 4E). DEGs were upregulated in circadian rhythm and downregulated in ribosome and type I diabetes mellitus (Figure 4F). Venn diagram showed that 57 common DEGs existed for WAT and BAT, but were not enriched for any signaling pathways (Supplementary Figure S3 and Supplementary Table S5).

**Figure 5:**
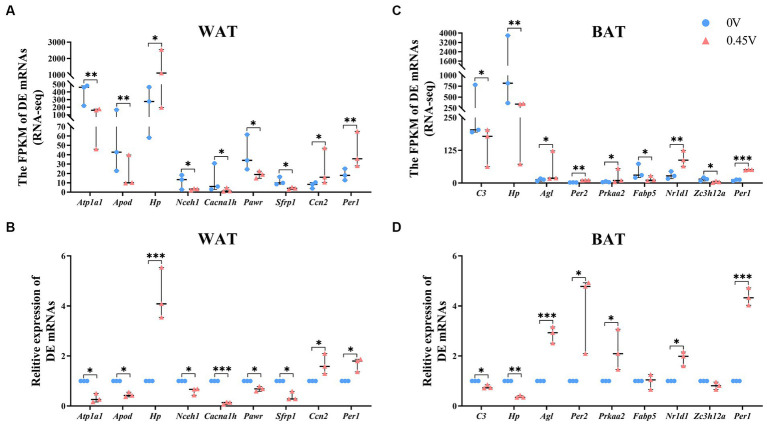
Transcriptional profiling of DEGs in white and brown adipose tissues. RNAseq and transcriptional validation of DEGs, for **(A,B)** WAT; **(C,D)** BAT, respectively. ^*^p < 0.05, ^**^p < 0.01, ^***^p < 0.001.

To validate RNAseq results, nine DEGs were selected for WAT [ATPase Na+/K+ transporting subunit α 1 (*Atp1a1*), apolipoprotein D (*Apod*), haptoglobin (*Hp*), neutral cholesterol ester hydrolase 1 (*Nceh1*), calcium voltage-gated channel subunit α 1 H (*Cacna1h*), PRKC apoptosis WT1 regulator (*Pawr*), secreted frizzled-related protein 1 (*Sfrp1*), cellular communication network factor 2 (*Ccn2*) and period circadian clock 1 (*Per1*)], and BAT [complement component 3 (*C3*), *Hp*, amylo-1, 6-glucosidase, 4-α-glucanotransferase (*Agl*), *Per2*, protein kinase, AMP-activated, α 2 catalytic subunit (*Prkaa2*), fatty acid binding protein 5, epidermal (*Fabp5*), nuclear receptor subfamily 1, group D, member 1 (*Nr1d1*), and zinc finger CCCH type containing 12A (*Zc3h12a*) and *Per1*]. For WAT, all nine DEGs had similar expression patterns to those of RNAseq (Figures 5A,B). For BAT, seven DEGs showed the same pattern, except for *Fabp5* and *Zc3h12a* (Figures 5C,D).

### Combined metagenomics and metabolomics analysis

**Figure 6:**
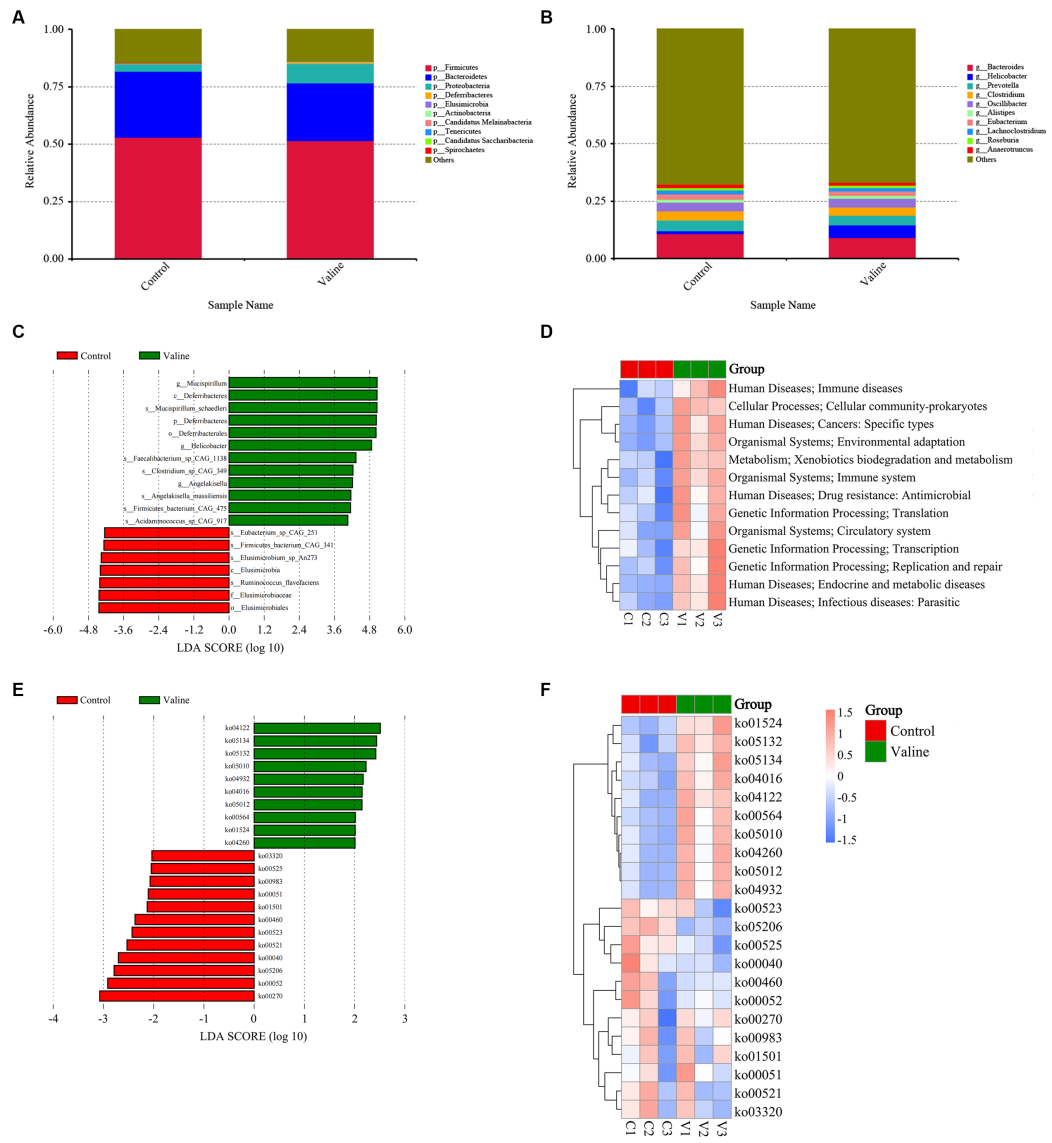
Gut metagenome sequencing. Dynamic changes of different proportions of microbe at the levels of **(A)** phylum; **(B)** genus; **(C)** LDA of different microbes; **(D)** heat map of KEGG signaling pathways (level 2); **(E)** LEfSe analysis identified 22 tertiary signaling pathways (LDA Score ≥ 2); **(F)** Signaling pathways enriched.

Gut microbiota is inextricably linked to obesity and IR (29), and we found that valine induced insulin insensitivity, disrupted intestinal and hepatic tissue structure and enhanced lipid deposition. Thus, we examined the cecal microbial composition and metabolites by integrating metagenome sequencing and untargeted metabolomics. First, PCoA clearly clustered the 0 V and 0.45 V samples into two distinct groups (Supplementary Figure S4A). Two groups had 992,469 common genera (Venn diagram), and 82,151 and 101,009 were specific to the 0 V and 0.45 V groups, respectively (Supplementary Figure S4B). Relative microbial abundance showed that at the phylum level, 0.45% valine reduced the proportion of *Bacteroidetes*, but increased that of *Proteobacteria* (*p* < 0.05) (Figure 6A); and at the genus level, *Bacteroidetes* and *Helicobacter* were reduced and increased, respectively (*p* < 0.05) (Figure 6B).

After microbial linear discriminant analysis (LDA) (Figure 6C) and KEGG pathway analysis (Figure 6D), the endocrine and metabolic diseases secondary signaling pathway and the immune system pathway in human disease were found to be enriched. Moreover, after LEfSe analysis, 22 tertiary signaling pathways (LDA Score ≥ 2) (Figure 6E) were found, including non-alcoholic fatty liver disease (ko04932), MAPK (ko04016), and glycerophospholipid metabolism (ko00564), as well as the neural system degeneration (ko05010 and ko05012) (Figure 6F).

**Figure 7:**
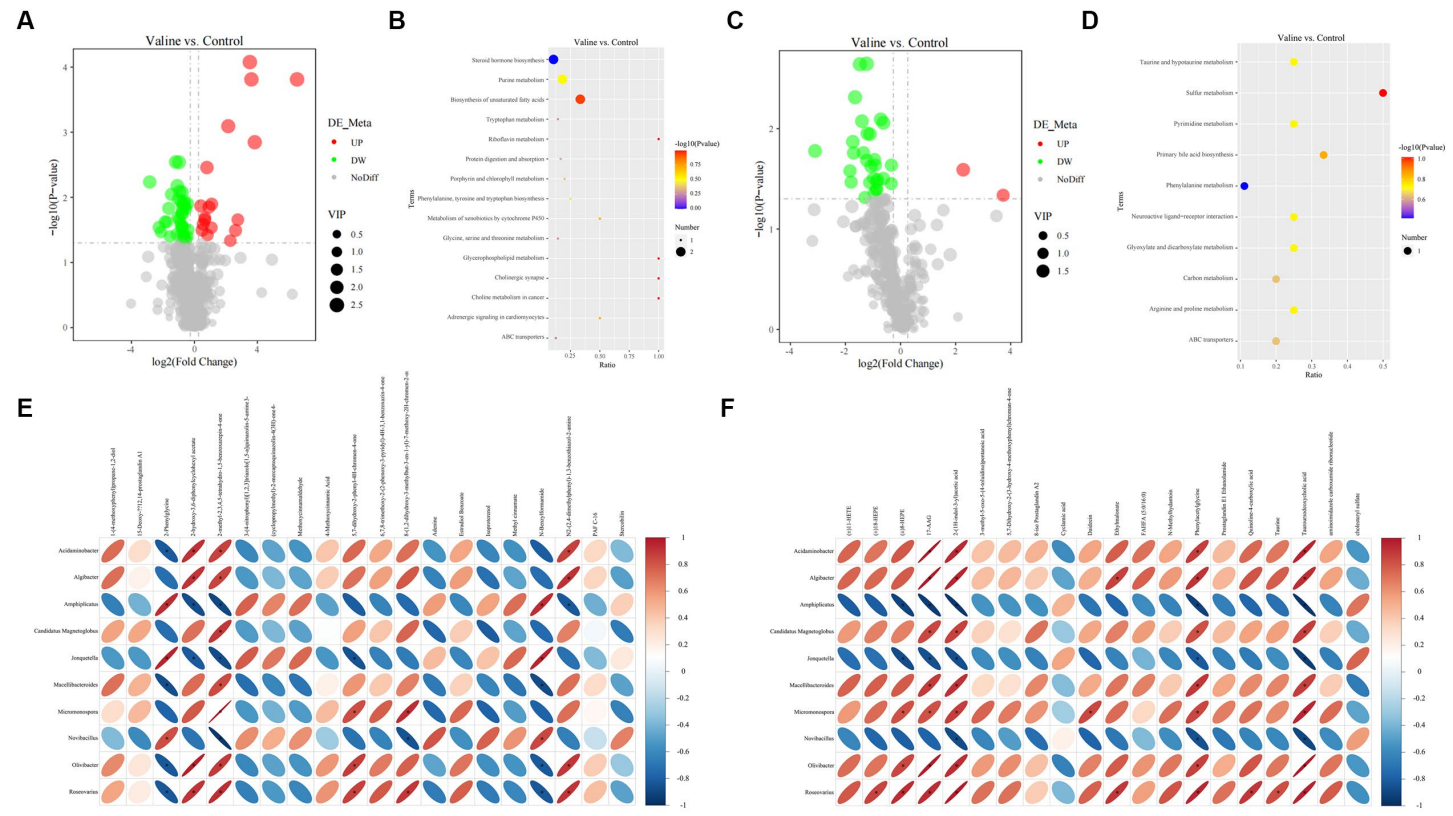
Gut metabolome and integrative analysis. **(A,C)** Volcano plots of differential metabolites (positive and negative modes); **(B,D)** enriched signaling pathways. Integrative analysis: **(E,F)** correlation between significant metabolites and microbes (positive and negative modes). Blue and red colors indicate negative and positive correlations, respectively. ^*^p < 0.05.

Non-targeted metabolomics was performed to observe how valine modulated intestinal metabolism. PCA did not separate samples into two different groups (Supplementary Figures S5A,D), but after the PLS-DA analysis, for both positive and negative modes, the 0 V and 0.45 V groups were clearly separated (Supplementary Figures S5B,E), and the model for the relationship between the metabolite and sample class was not over-fit (*R*^2^ > *Q*^2^, bias <0) (Supplementary Figures S5C,F). After differential analysis (criteria: VIP > 1.0 and FC > 1.2, or FC < 0.833 and *p* < 0.05), 87 metabolites (20 up- and 67 down-regulated) were identified (Figures 7A,C and Supplementary Figure S6). Enrichment analysis based on differential metabolites showed that multiple pathways, such as protein digestion and absorption, and those related to lipid metabolism (steroid hormone biosynthesis, biosynthesis of unsaturated fatty acids, glycerophospholipid metabolism, and primary bile acid biosynthesis) were affected (Figures 7B,D).

Further combined metagenome and metabolome analysis showed the correlation between microbes (top 10 genera) and differential metabolites (positive and negative) (top 20, starting from the largest |log2(FoldChange)|), respectively (Figures 7E,F). Interestingly, there existed similar strong correlation patterns between two metabolites (17-AAG and tauroursodeoxycholic acid) and different microbes (Figure 7F).

### Valine dysregulates inflammation and lipid metabolism pathways

**Figure 8:**
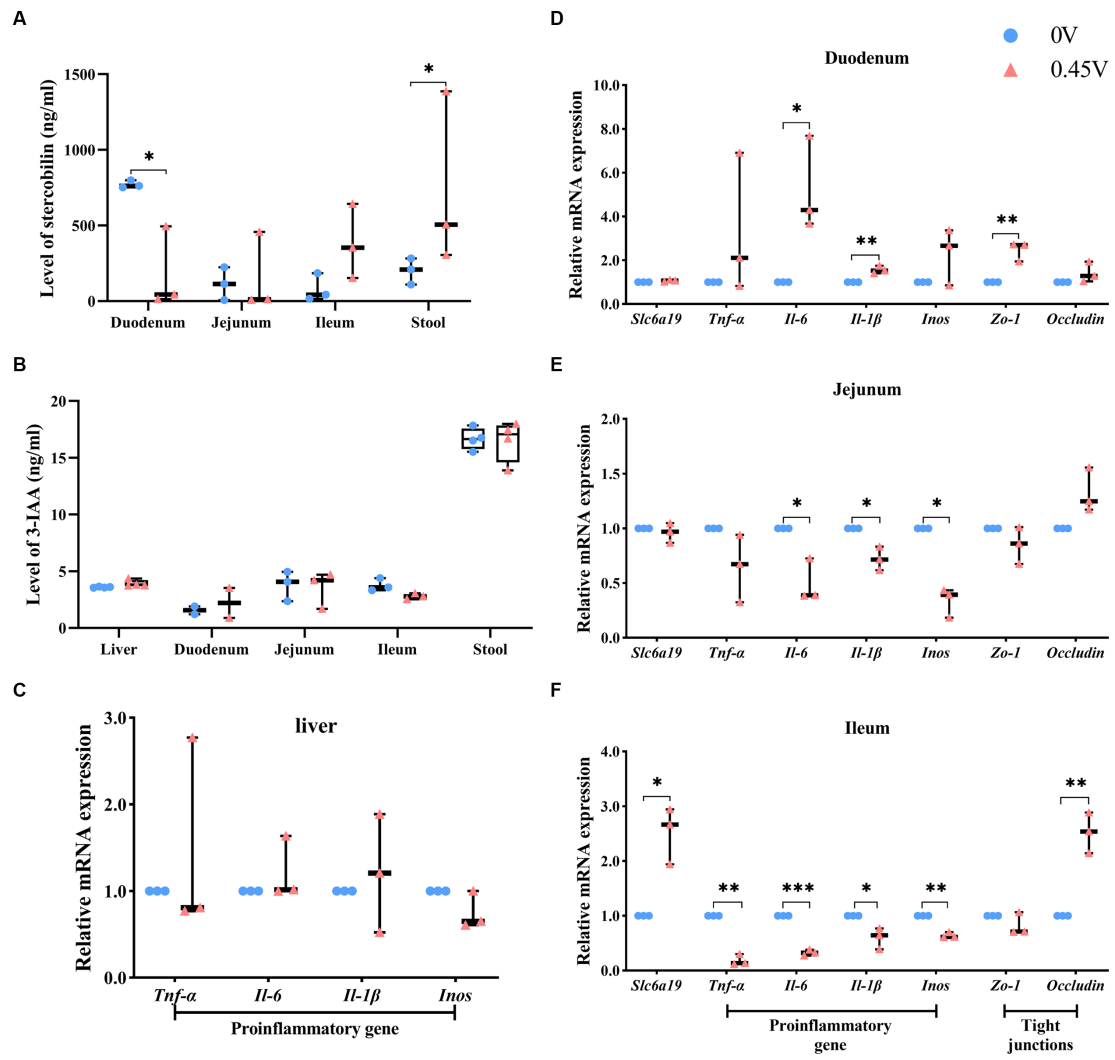
Validation of metabolites and the inflammation pathway. Two metabolites: **(A)** stercobilin; **(B)** 3-IAA. Expression profiling on genes related to inflammation and gap junction: **(C)** liver; **(D)** duodenum; **(E)** jejunum; **(F)** ileum. ^*^p < 0.05, ^**^p < 0.01, ^***^p < 0.001.

Combined RNAseq, metagenomics, and metabolomics analysis highlighted the fact that inflammation and lipid metabolism pathways in the small intestine and metabolic tissues could be affected by valine supplementation. The intestinal microbial metabolite, stercobilin, previously reported to be associated with chronic inflammation (40), was significantly altered by valine treatment. Therefore, we assayed its level and that of another metabolite (3-IAA) by ELISA. In duodenum and stool, stercobilin was significantly inhibited and elevated by valine treatment (0.45 V), respectively (*p* < 0.05) (Figure 8A). Stercobilin content in stool was consistent with the metabolomics result. In the ileum, stercobilin was also enhanced, though not significantly different. However, 3-IAA was not significantly different (Figure 8B).

In addition, expression levels of inflammation-related genes were evaluated in the liver. However, no significant difference was found (Figure 8C). Furthermore, we profiled the expression levels of genes related to neutral amino acid transferring [solute carrier family 6 (neurotransmitter transporter), member 19 (*Slc6a19*)], inflammation, and gap junction in the small intestine (the duodenum, the jejunum, and the ileum). With 0.45% valine supplementation, inflammation genes were increased significantly in the duodenum [interleukin 6 (*Il-6*), *p* < 0.05; interleukin 1β (*Il-1β*), *p* < 0.01] (Figure 8D). However, in the jejunum, they were inhibited significantly [*Il-6*, *Il-1β*, and nitric oxide synthase 2, inducible (*Inos*) was, *p* < 0.05] (Figure 8E), and especially in the ileum, which is the most significantly inhibited [tumor necrosis factor α (*Tnf-α*), *p* < 0.01; *Il-6*, *p* < 0.001; *Il-1β*, *p* < 0.05 and *Inos*, *p* < 0.01] (Figure 8F).

Furthermore, gap junction genes, tight junction protein 1 (*Zo-1*) and *Occludin*, were enhanced in the duodenum and ileum (*p* < 0.01) of the 0.45 V group, respectively (Figures 8D,F). Expression of *Slc6a19* only increased in the ileum (*p* < 0.05) of the 0.45 V group (Figure 8F).

## Discussion

As each member of BCAAs is found to play either a beneficial or detrimental role to host metabolism (15–22), in the current study, we aimed to examine the specific role of valine in metabolic health. Here, by a multi-Omics approach, we showed that valine can disturb the cecal microbiota and metabolite compositions, with *Bacteroidetes* decreased, but *Proteobacteria* and *Helicobacter* increased. The inflammation in the duodenum was induced, and lipid metabolism in hepatic and adipose tissues was also enhanced.

Originally, valine was discovered to be related to BAT thermogenesis and affected glucose intake and IR of metabolic diseases (19, 20). The metabolite product of valine, 3-HIB, was elevated and considered a circulating biomarker in obese and diabetic patients (12, 14). We found that reduced insulin sensitivity was incurred, and hepatic TG and TC levels were found to be increased by 0.30% post valine treatment. A recent study also showed that in pig intestinal epithelial cells (IPEC-J2), valine and 3-HIB promoted fatty acid transport and enhanced TG synthesis, lipid droplet formation, and unsaturated fatty acid concentration (41). We further demonstrated that hepatic *de novo* lipogenesis and adipogenesis in WAT were increased, and β-oxidation in WAT and BAT was inhibited by valine. Thus, valine and 3-HIB could be directly involved in the enhancement of fatty acid and lipid metabolism.

The influence of BCAAs on individual metabolism is closely related to the gut microbiome. Studies have shown that the gut microbiome in insulin-resistant individuals has more *Prevotella copri* and *Bacteroides vulgatus* species, with the tendency to synthesize more BCAAs (28, 42, 43). *P. copri* was demonstrated to be capable of inducing IR, aggravating glucose intolerance and augmenting circulating levels of BCAAs (28). In addition, BCAA catabolism in BAT is important for alleviating IR (19, 20). Feeding *Bacteroides dorei* and *vulgatus* to mice improved BAT BCAA catabolism, decreased circulating BCAA levels and body weight gain (44). Moreover, *Bacteroides* could be used as probiotics for treating aberrant metabolism, which is currently under active research (44–46). We found here that *Bacteroides* decreased in cecum, which could be related to the detrimental effect of valine on lipid metabolism in hepatic and adipose tissues.

Further assessment revealed that valine increased inflammation and reduced crypt depth in the duodenum. On the contrary, it seems that valine could be absorbed mainly and reduce inflammation in the ileum, as indicated by the local high expression levels of *Slc6a19* and reduced expression of inflammation-related genes (*Tnf-α* and interleukins) (40). Moreover, the structural integrity of the small intestine and systemic inflammation are reported to be linked to IR (40, 43). Adipose tissue transcriptome sequencing also indicated the existence of adipose inflammation. Signaling pathways related to lipid metabolism and adipogenesis were also found to be disturbed by valine supplementation, including bile acid and circadian rhythm. Interestingly, the core circadian clock member *Per1* regulates directly the phosphorylation of bile acid synthases through PKA and, in turn, affects fat absorption and accumulation (47). *Per1* also interacts with GPX1 to mediate mitochondrial function and metabolic homeostasis in response to the oxidation process (48). Moreover, intestinal metabolic dysregulation and hepatic inflammation could be induced by disrupted circadian rhythmic stasis (49). It is worthwhile to investigate further how valine affects the molecular clock and metabolic health.

Microbes directly digest, absorb, and metabolize food nutrients and produce metabolites to affect inter-organ and holistic metabolism (50–54). Among them, stercobilin is the metabolite directly involved in bile acid metabolism and associated with chronic inflammation of obese mice (40). Here, valine induced the increased stercobilin level in cecal content, which could contribute to inflammation and enhanced lipid metabolism, as observed in small intestine and adipose tissues. The diet, metabolite, and microbiota axis is of functional importance to human health, as also shown previously by a multi-omic analysis (53). Furthermore, in the present study, valine was supplemented via drinking water. Nowadays, functional beverages, containing different bioactive substances (for instance, BCAAs and vitamins in sports drinks), are consumed daily by humans (55, 56). However, valine can have adverse effects on metabolic health as found here and reproductive function as reported previously (23), serving as a caution to its use in functional drinks. Certainly, our study is limited, and a detailed investigation is warranted to understand the exact role and effect of valine on human metabolic health.

Taken together, the multi-Omics analysis revealed that valine treatment enhances hepatic lipid metabolism and adipogenesis, by affecting the intestinal microbiota and inflammation in gut and adipose tissues. Our findings provide novel insights and resources for further investigation on the molecular mechanism and biological function of valine on lipid metabolism.

## Data availability statement

The data presented in the study are deposited in the NCBI repository, accession number SRR27778108–SRR27778119, SRR28172663–SRR28172668.

## Ethics statement

The animal study was approved by Animal Research Committee guidelines of Yangtze University. The study was conducted in accordance with the local legislation and institutional requirements.

## Author contributions

H-YZ: Data curation, Formal analysis, Methodology, Validation, Writing – original draft. LW: Data curation, Formal analysis, Methodology, Validation, Writing – original draft. RZ: Software, Writing – original draft. RD: Methodology, Writing – original draft. C-XY: Funding acquisition, Methodology, Project administration, Supervision, Writing – original draft, Writing – review & editing. Z-QD: Funding acquisition, Methodology, Project administration, Supervision, Writing – original draft, Writing – review & editing.

## Footnotes

^1^ http://www.ensembl.org/index.html

^2^ https://www.genome.jp/kegg/pathway.html

^3^ https://hmdb.ca/metabolites

^4^ http://www.lipidmaps.org/

## Supplementary material

### SUPPLEMENTARY FIGURE S1

Valine treatment on mice growth and metabolism. **(A)** Body weights; **(B)** weight gains; **(C)** feed intake; **(D)** water intake; **(E)** glucose tolerance test (GTT); **(F)** pyruvate tolerance test (PTT); **(G)** insulin tolerance test (ITT). ^*^*P* < 0.05, ^**^*P* < 0.01.

### SUPPLEMENTARY FIGURE S2

Transcriptome sequencing on adipose tissues. Transcriptional dynamics of genes for **(A)** White adipose tissue (WAT); **(B)** brown adipose tissue (BAT); PCA sample clustering for **(C)** WAT; **(D)** BAT.

### SUPPLEMENTARY FIGURE S3

Venn diagram of common differentially expressed genes (DEGs) for WAT and BAT.

### SUPPLEMENTARY FIGURE S4

Cecal metagenome sequencing. **(A)** Principal co-ordinates analysis (PCoA); **(B)** Venn diagram.

### SUPPLEMENTARY FIGURE S5

Non-targeted metabolomics. For the positive and negative modes, **(A,D)** PCA clustering; **(B,E)** PLS-DA analysis; **(C,F)** The fitted model parameters.

### SUPPLEMENTARY FIGURE S6

Metabolites significantly different. **(A)** Positive mode; **(B)** negative mode.
