# Supplementary figures and images for: Valine induces inflammation and enhanced adipogenesis in lean mice by multi-omics analysis
Source: Front Nutr. 2024 May 13;11:1379390. doi: 10.3389/fnut.2024.1379390 (PMC11128663; doi:10.3389/fnut.2024.1379390)

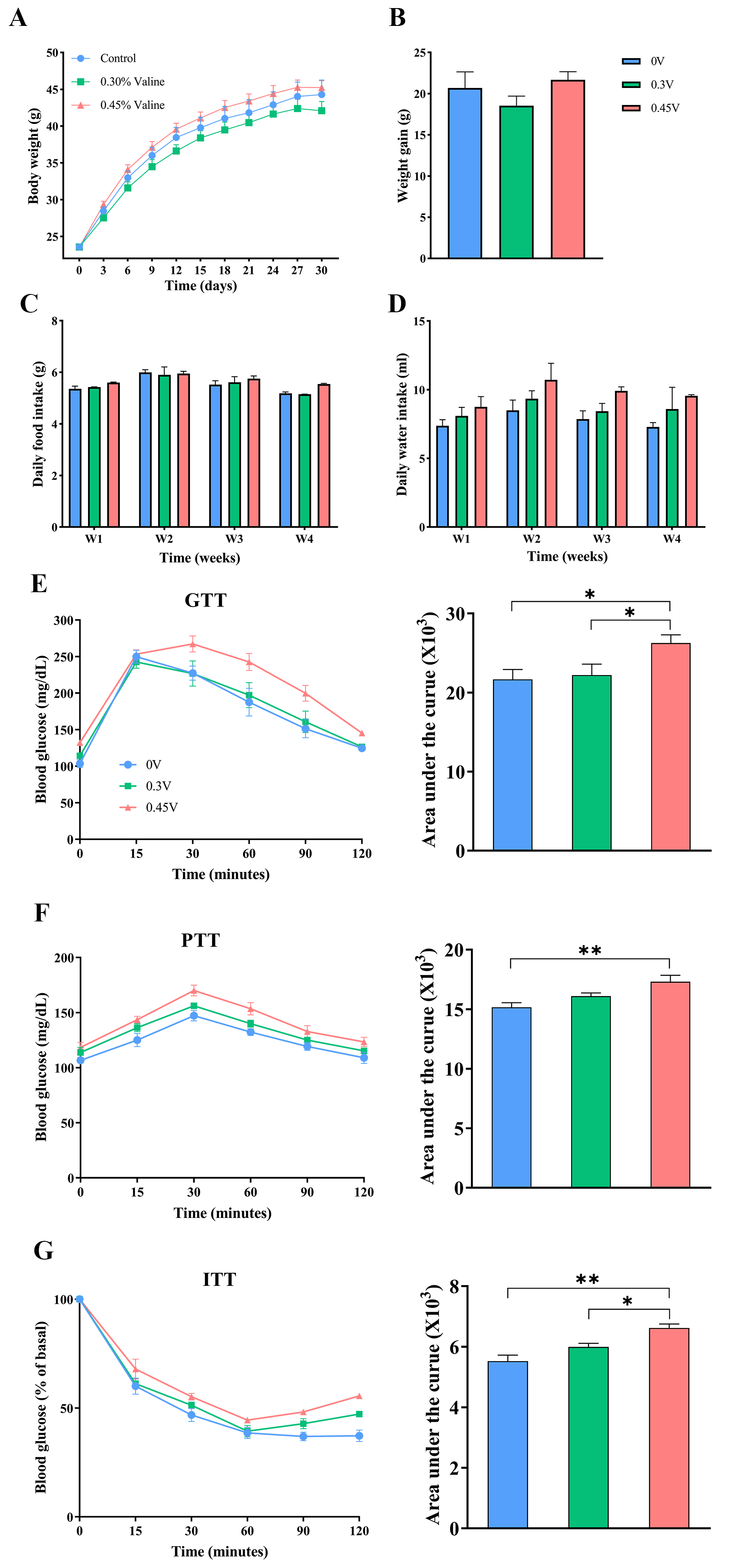

Supplement: Supplementary file 1 [file Image_1.TIF]

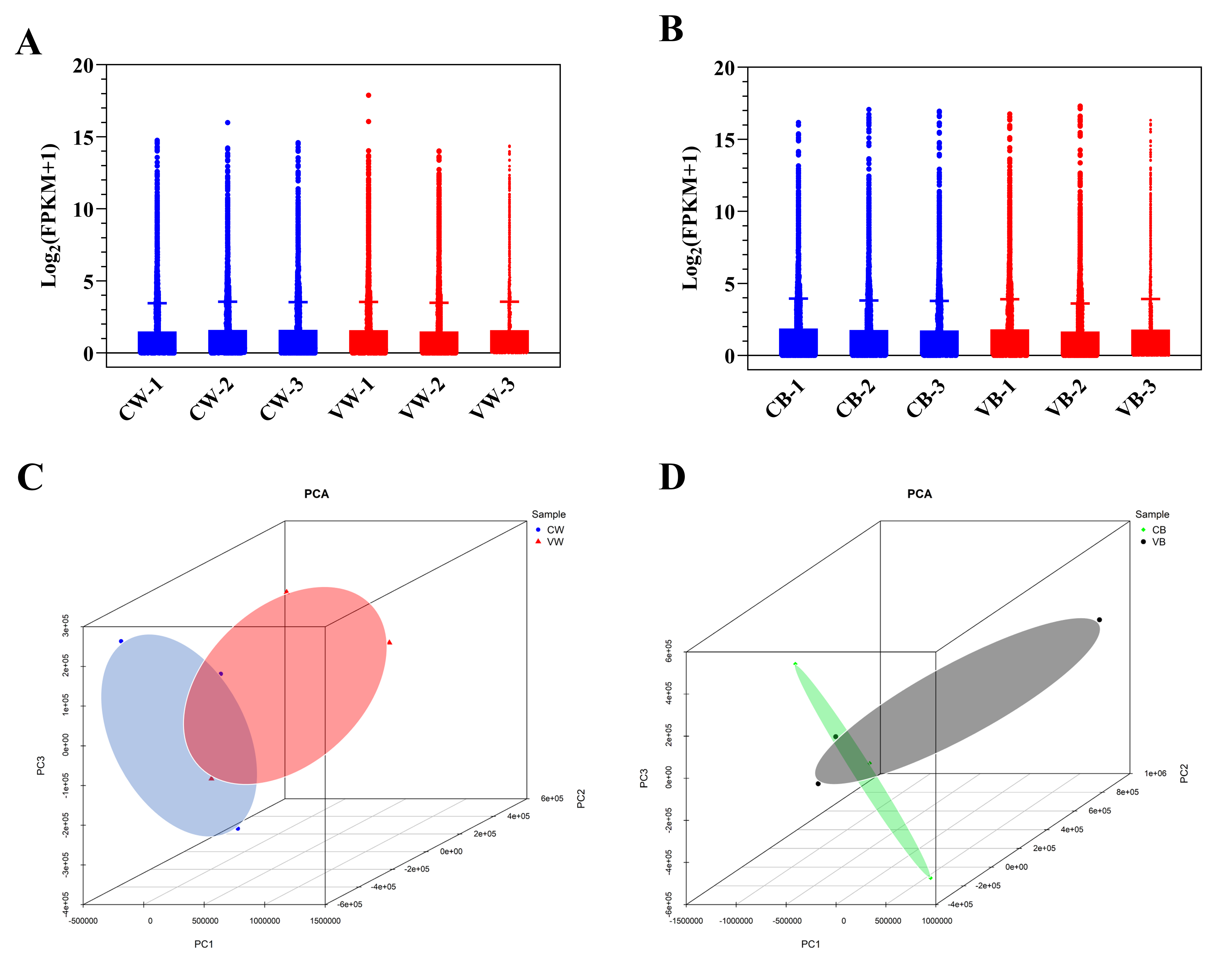

Supplement: Supplementary file 2 [file Image_2.TIF]

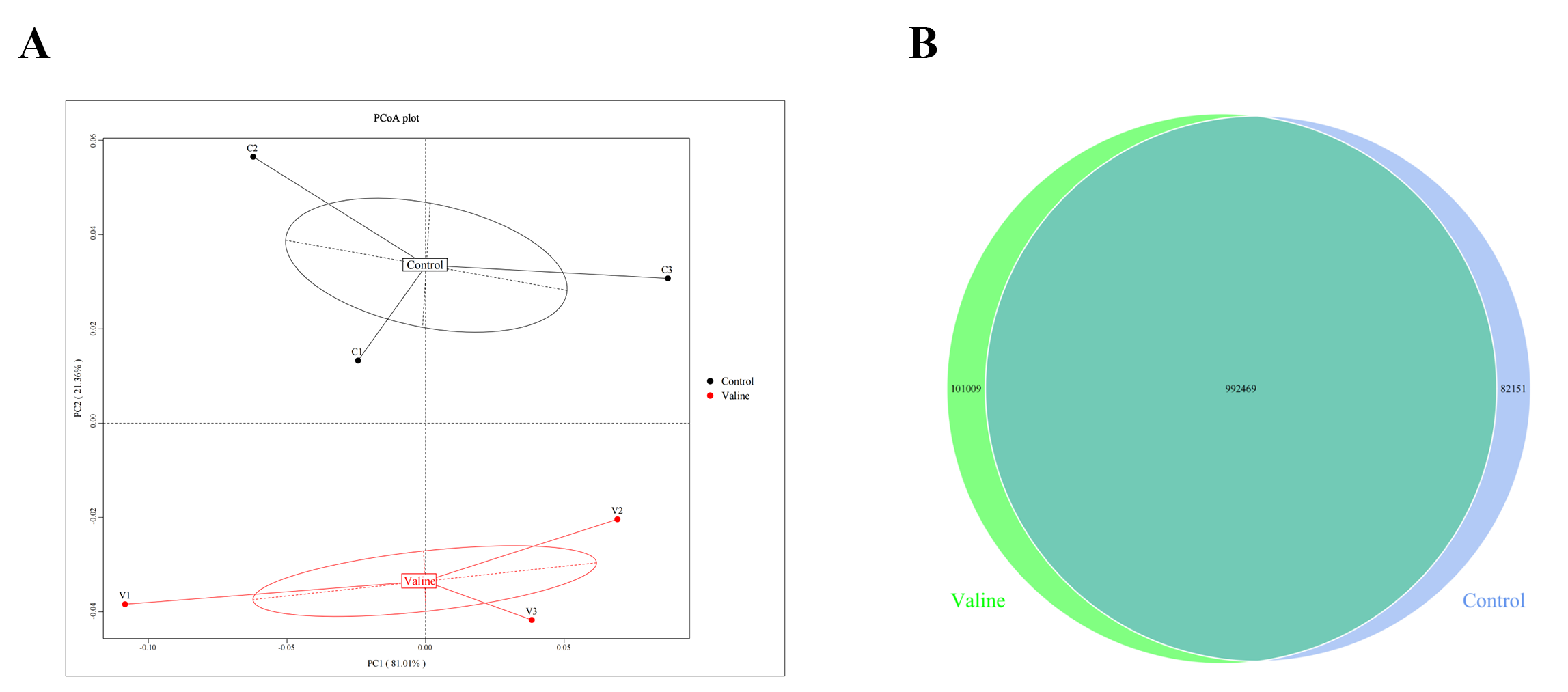

Supplement: Supplementary file 4 [file Image_4.TIF]

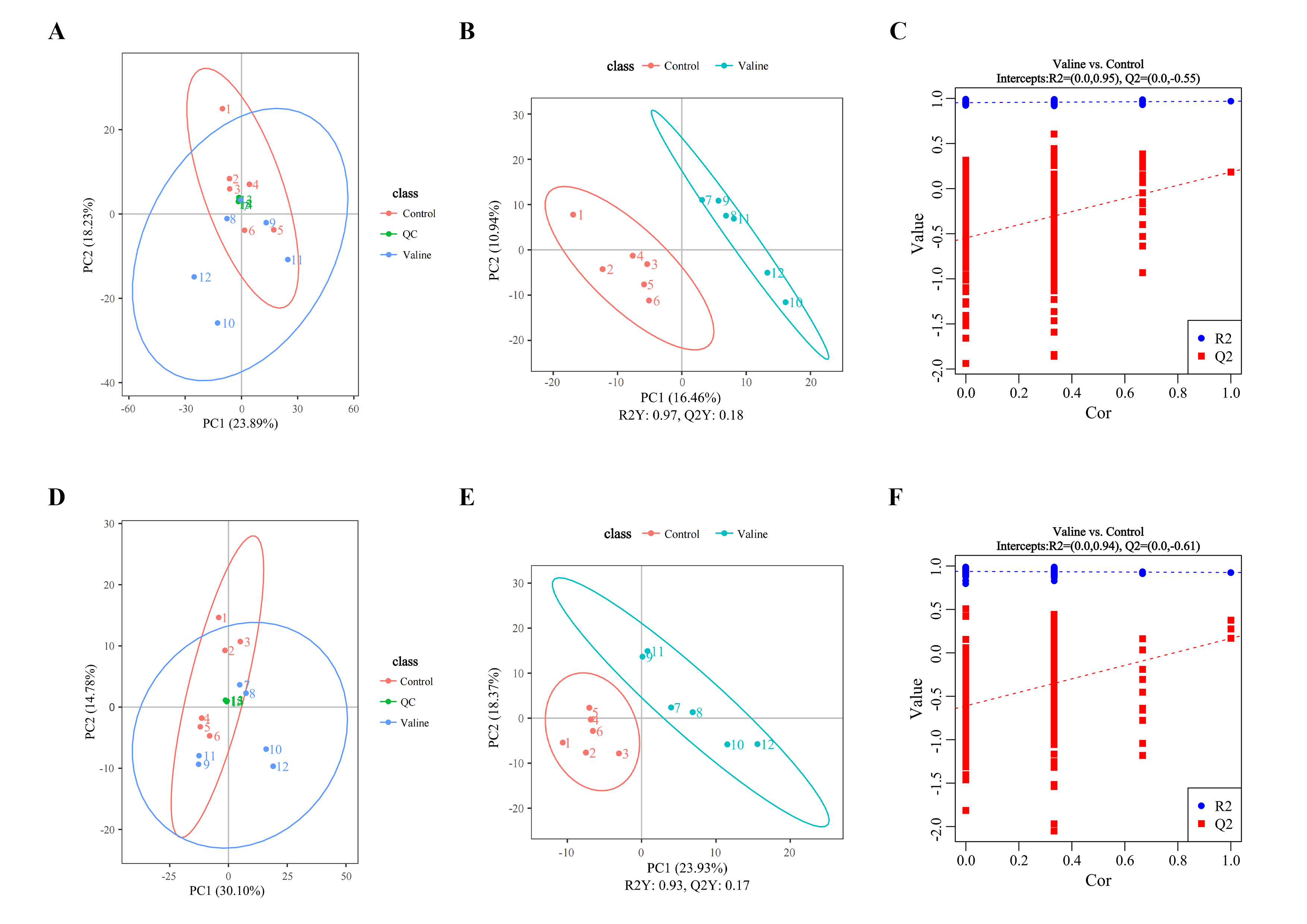

Supplement: Supplementary file 5 [file Image_5.TIF]

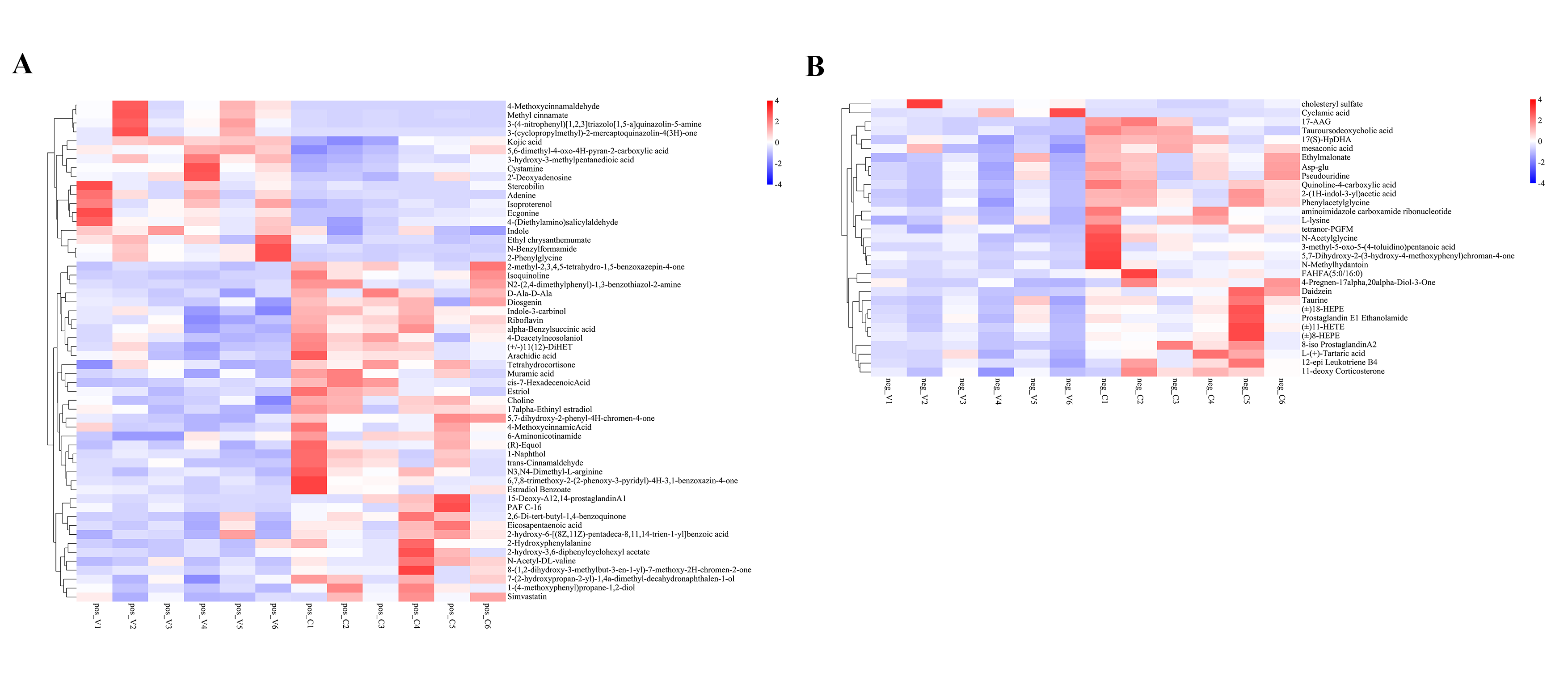

Supplement: Supplementary file 6 [file Image_6.TIF]
