# Supplementary material for: Valine induces inflammation and enhanced adipogenesis in lean mice by multi-omics analysis
Source: Front Nutr. 2024 May 13;11:1379390. doi: 10.3389/fnut.2024.1379390 (PMC11128663; doi:10.3389/fnut.2024.1379390)
Supplement: Supplementary file 7 [file Table_1.DOCX]

**Table S1 Nutrient composition per kilogram of feed for mice fodder**

| Ingredient | Content (1Kg Fodder) |
| --- | --- |
| Moisture and other volatile substances | ≤100g |
| Crude protein | ≥200g |
| Crude fat | ≥40g |
| Crude fibre | ≤50g |
| Coarse ash | ≤80g |
| Ca | 10—18g |
| P | 6—12g |
| Ca: P | 1.2：1—1.7:1 |
| Lysine | ≥13.2g |
| Methionine Cystine | ≥7.8g |
| Arginine | ≥11.0g |
| Histidine | ≥5.5g |
| Tryptophan | ≥2.5g |
| Phenylalanine Tyrosine | ≥13.0g |
| Threonine | ≥8.8g |
| Leucine | ≥17.6g |
| Isoleucine | ≥10.3g |
| Valine | ≥11.7g |
| Vitamin A | 14000IU |
| Vitamin D | 1500IU |
| Vitamin E | 120IU |
| Vitamin K | 5.0mg |
| Vitamin B_1_ | 13mg |
| Vitamin B_2_ | 12mg |
| Vitamin B_6_ | 12mg |
| Nicotinic acid | 60mg |
| Acid regurgitatio | 24mg |
| Folic acid | 6.00mg |
| Biotin | 0.20mg |
| Vitamin B_12_ | 0.022mg |
| Choline | 1250mg |
| Mg | 2.0mg |
| K | 5.0mg |
| Na | 2.0mg |
| Fe | 120mg |
| Mn | 75mg |
| Cu | 10mg |
| Zn | 30mg |
| I | 0.5mg |
| Se | 0.1-0.2mg |
