# Supplementary material for: Valine induces inflammation and enhanced adipogenesis in lean mice by multi-omics analysis
Source: Front Nutr. 2024 May 13;11:1379390. doi: 10.3389/fnut.2024.1379390 (PMC11128663; doi:10.3389/fnut.2024.1379390)
Supplement: Supplementary file 8 [file Table_2.DOCX]

**Table S2 Quality control of RNA samples.**

| Sample | Density (ng/μL) | Volume (μL) | RNA content (ug) | Integrity |
| --- | --- | --- | --- | --- |
| CW1 | 267.000 | 7.30 | 1.94910 | 4.50 |
| CW2 | 323.000 | 16.00 | 5.16800 | 6.90 |
| CW3 | 188.000 | 7.40 | 1.39120 | 5.20 |
| VW1 | 356.000 | 16.00 | 5.69600 | 7.10 |
| VW2 | 290.000 | 4.60 | 1.33400 | 5.30 |
| VW3 | 345.000 | 16.00 | 5.52000 | 5.90 |
| CB1 | 408.000 | 16.00 | 6.52800 | 4.10 |
| CB2 | 326.000 | 17.00 | 5.54200 | 7.00 |
| CB3 | 309.000 | 17.00 | 5.25300 | 7.00 |
| VB1 | 586.000 | 17.00 | 9.96200 | 6.80 |
| VB2 | 371.000 | 17.00 | 6.30700 | 7.30 |
| VB3 | 263.000 | 16.00 | 4.20800 | 6.80 |
