# Supplementary material for: Valine induces inflammation and enhanced adipogenesis in lean mice by multi-omics analysis
Source: Front Nutr. 2024 May 13;11:1379390. doi: 10.3389/fnut.2024.1379390 (PMC11128663; doi:10.3389/fnut.2024.1379390)
Supplement: Supplementary file 9 [file Table_3.DOCX]

**Table S3 Primers used for qRT-PCR**

| Gene | Forward (5'-3') | Reverse (5'-3') | Size (bp) |
| --- | --- | --- | --- |
| Srebp-1c | GGGGCCTGACAGGTGAAATC | TGAGCTGGAGCATGTCTTCAAA | 116 |
| Cebpα | TTCGGGTCGCTGGATCTCTA | TCAAGGAGAAACCACCACGG | 164 |
| Fas | GTCAACCATGCCAACCTGAAAAC | GTTTGGCTTCTTTACCCACCC | 139 |
| Scd1 | CTTATCATTGCCAACACCA | CAAGCAGCCAACCCAC | 148 |
| Acaca | TCTATCCGTCGGTGGT | GAGCAGTTCTGGGAGTTT | 261 |
| Lpl | CATGGATGGACGGTAACGGG | TGGACGTTGTCTAGGGGGTA | 224 |
| Acox1 | GCCGTCGAGAAATCGAGAAC | GAGGCCAACAGGTTCCACAA | 212 |
| Pparα | CAGATTCCGCCTGTCCGTC | CCACAGAGCGCTAAGCTGTGA | 238 |
| Cpt1 | TCTCCATGGGACTGGTCGAT | GAGACGGACACAGATAGCCC | 239 |
| Atp1a1 | CAAGGAGCAGCCACTGGATGAAG | ACTGTTCGTCAGGCAGGAGGAG | 119 |
| APod | ATGCCCGTCTCCTCCTGTGC | CCGTTCTCCATCAGCGAGTAGTTG | 135 |
| Hp | GCTGTTGTCACTCTCCTGCTCTG | CGGCAGCGATAGCGAACCAAG | 143 |
| Nceh1 | TCCCTGTTGCCCTCGTCCATC | CAGCAGCGTCCAGCAGTTGAG | 103 |
| Cacna1h | GCCTTTCTCAGCGTCTCCAACTAC | GCAGCCCGTCCAGCACATTC | 136 |
| Pawr | GCGGAGTGCTTAGATGAGTACGAAG | TGGATGGTGTTTTGCTGGGTGATG | 89 |
| Sfrp1 | CATGGCAGAGGTGAAGCAGCAG | GCGAAGAGCGAACAGAGGAAGAC | 96 |
| Ccn2 | CACCGCACAGAACCACCACTC | AATGGCAGGCACAGGTCTTGATG | 99 |
| Per1 | CCTGGGCTCTGGGTCTGGTTC | TTGCTTGTATGGCTGCTCTGACTG | 90 |
| C3 | GTTCGGCATAGAGAAGAGGCAAGAG | TTGTTGAAGGCAGCATAGGCAGAG | 105 |
| Agl | GCTGCTGGTTACTGGACGCTAC | TTCGCCGAGGAGGTTAGGGATG | 97 |
| Per2 | GCTGCGGATGCTCGTGGAATC | GGTTGTGCTCTGCCTCTGTCATC | 86 |
| Prkaa2 | ATGATGAGGTGGTGGAGCAGAGG | AGTGAGAGAGCCAGAAAGGGAGTG | 134 |
| Fabp5 | GCTAGGAGTAGGACTGGCTCTTAGG | TCTTCACTGTGCTCTCGGTTTTGAC | 110 |
| Nr1d1 | CGTCATCCTCTTCATCCTCCTCCTC | CTTGGTAATGTTGCTTGTGCCCTTG | 119 |
| Zc3h12a | TGCTGGCTGTGAACTGGTTTCTG | CGAAGGATGTGCTGGTCTGTGATAG | 118 |
| Slc6a19 | TCACGAAGATGCCAGTGTCC | CTGAAGGGGTACGACCACAC | 113 |
| Tnf-α | CCACCACGCTCTTCTGTCTACTG | GATGATCTGAGTGTGAGGGTCTGG | 118 |
| Il-6 | TTCTTGGGACTGATGCTGGTGAC | CTGTTGGGAGTGGTATCCTCTGTG | 89 |
| Il-1β | TGCCACCTTTTGACAGTGATG | TGATGTGCTGCTGCGAGATT | 138 |
| Inos | ATGACTCCCAGCACAAAGGG | CTCTCTTGCGGACCATCTCC | 117 |
| Zo-1 | GGAGATGTTTATGCGGACGG | TCCATTGCTGTGCTCTTAGCG | 137 |
| Occludin | GCCTACTCCTCCAATGGCAA | CATAGTGGTCAGGGTCCGTC | 229 |
| β-actin | TGGAATCCTGTGGCATCCATGAAAC | TAAAACGCAGCTCAGTAACAGTCCG | 349 |
