# Supplementary material for: Valine induces inflammation and enhanced adipogenesis in lean mice by multi-omics analysis
Source: Front Nutr. 2024 May 13;11:1379390. doi: 10.3389/fnut.2024.1379390 (PMC11128663; doi:10.3389/fnut.2024.1379390)
Supplement: Supplementary file 10 [file Table_4.DOCX]

**Table S4 Quality statistics for RNAseq short reads.**

| **Sample** | **Raw Reads** | **Clean Reads** | **Clean Base** | **Error (%)** | **Q20 (%)** | **Q30 (%)** | **GC (%)** |
| --- | --- | --- | --- | --- | --- | --- | --- |
| CW1 | 42628712 | 41665766 | 97.74 | 6.25 | 97.81 | 94.09 | 49.5 |
| CW2 | 42808038 | 41561602 | 97.09 | 6.23 | 97.9 | 94.24 | 50.06 |
| CW3 | 50005924 | 48895484 | 7.33 | 7.33 | 97.80 | 94.06 | 49.23 |
| VW1 | 40945544 | 40080674 | 97.89 | 6.01 | 97.74 | 93.84 | 52.49 |
| VW2 | 45309762 | 44431258 | 98.06 | 6.66 | 97.83 | 94.18 | 50.12 |
| VW3 | 51841980 | 50714482 | 97.83 | 7.61 | 97.93 | 94.39 | 49.48 |
| CB1 | 48841224 | 47561008 | 97.38 | 7.13 | 97.78 | 94 | 48.65 |
| CB2 | 53455172 | 52188720 | 97.63 | 7.83 | 97.83 | 93.96 | 47.15 |
| CB3 | 42152982 | 40446220 | 95.95 | 6.07 | 97.86 | 94.01 | 46.99 |
| VB1 | 43804664 | 43138812 | 98.48 | 6.47 | 97.53 | 93.25 | 47.93 |
| VB2 | 44163490 | 43568816 | 98.65 | 6.54 | 97.84 | 93.88 | 43.37 |
| VB3 | 45304038 | 44343818 | 97.88 | 6.65 | 97.93 | 94.15 | 49.6 |
