# Supplementary material for: Valine induces inflammation and enhanced adipogenesis in lean mice by multi-omics analysis
Source: Front Nutr. 2024 May 13;11:1379390. doi: 10.3389/fnut.2024.1379390 (PMC11128663; doi:10.3389/fnut.2024.1379390)
Supplement: Supplementary file 11 [file Table_5.DOCX]

**Table S5 Common differentially expressed genes (DEGs) between white and brown adipose tissues.**

| **Common DEG name** | **White adipose tissue** | **Brown adipose tissue** |
| --- | --- | --- |
|  | **\|log2FC\| ≥ 1，Pvalue < 0.05** | **\|log2FC\| ≥ 1，Pvalue < 0.05** |
| 6330409D20Rik | -2.156424289 | 2.819408626 |
| 9330159F19Rik | -1.597311005 | 4.130039232 |
| Acmsd | -4.082925141 | 2.74359872 |
| Adamts8 | 4.019289051 | 2.678717844 |
| Agbl2 | 3.989923769 | 2.632621793 |
| Arrdc3 | -1.334662079 | 1.472767967 |
| Camk4 | 3.48068673 | -5.206300757 |
| Ccdc146 | 4.421078261 | 2.680749534 |
| Ccn2 | -1.69771603 | 2.186190878 |
| Clip4 | 1.817652328 | 2.797544296 |
| Dchs2 | 2.656346906 | 2.962550282 |
| Dpep2 | -3.614999358 | -4.809405516 |
| Dtx1 | 3.085679708 | -2.226359845 |
| Uckl1os | 3.56200116 | 2.87085485 |
| Gm5424 | 2.15705671 | -2.960901122 |
| Rpsa-ps10 | -2.86419793 | 2.700747199 |
| Rps3a3 | 2.497278466 | -3.525391011 |
| Rps3a2 | 4.605099438 | -4.473378721 |
| ENSMUSG00000081671 | -2.17049882 | -4.583319122 |
| ENSMUSG00000083209 | -3.302471848 | 3.162859321 |
| 4930511M06Rik | 2.470404715 | 3.093444495 |
| ENSMUSG00000089656 | -4.481679754 | 4.674444923 |
| Gm5627 | -1.336737002 | -3.299535498 |
| Gm9625 | -6.495479999 | 4.966986716 |
| ENSMUSG00000102917 | 2.248473536 | -2.937733797 |
| ENSMUSG00000107620 | -4.542692648 | 4.482726843 |
| ENSMUSG00000108068 | -1.312205022 | -2.310694109 |
| ENSMUSG00000109555 | -1.657646916 | 2.657567857 |
| Gm7600 | 3.231117608 | -4.945739795 |
| Gm18258 | -2.430518132 | 2.131106798 |
| ENSMUSG00000117628 | 1.437950468 | -4.038090637 |
| ENSMUSG00002075736 | -1.547741846 | 1.850217177 |
| Espl1 | -1.090840979 | -1.909387218 |
| Eya4 | 2.902760482 | 2.803427121 |
| Fam167a | 4.596641156 | 1.501253812 |
| Fbxl16 | 2.620399391 | 1.691232481 |
| Galnt17 | 2.287771574 | 2.540321606 |
| Homer2 | 2.978583238 | 2.773570003 |
| Hp | -2.291441867 | -2.755860251 |
| Kcnab1 | 1.731653745 | 2.420510855 |
| Kif1a | 2.651252564 | -1.871844801 |
| Lvrn | -1.167907272 | 1.314693615 |
| Ly6d | 2.541364344 | -4.238383587 |
| Mpp4 | 2.275669445 | -2.962588163 |
| Nek10 | 3.970841917 | 4.146806203 |
| Paqr5 | 2.831039376 | -4.23667847 |
| Per1 | -1.204556421 | 2.161011689 |
| Pknox2 | 1.472801143 | 2.263408995 |
| Rasd2 | 1.926378452 | 2.085572236 |
| Sh2d5 | -1.925365276 | 1.665106325 |
| Sobp | 2.215645922 | 2.755188731 |
| Sorbs2 | 1.871046965 | 2.245044173 |
| Tekt1 | 3.662841427 | 2.570893835 |
| Tent5b | 1.868083936 | 2.329975207 |
| Tnfrsf8 | 2.37064535 | -4.216442759 |
| Tspoap1 | 2.409967714 | -5.335743367 |
| Ttll7 | 2.633743729 | 3.599628263 |
